# Continuous PPI Treatment After Gastric Bypass Increases the Risk of Pathological PTH Levels at 10 Years Postoperatively

**DOI:** 10.1007/s11695-025-07692-0

**Published:** 2025-01-27

**Authors:** Katharina Stevens, Hella Hultin, Magnus Sundbom

**Affiliations:** 1 Uppsala University Hospital, Uppsala, Sweden; 2 Karolinska Institutet, Stockholm, Sweden

**Keywords:** Gastric bypass, Bariatric surgery, Parathyroid hormone, Proton-pump inhibitors

## Abstract

**Background:**

Apart from massive weight loss, metabolic and bariatric surgery, especially gastric bypass (Roux-en-Y gastric bypass [RYGB]), can cause nutritional deficiencies. Proton pump inhibitors (PPI), relatively often used after RYGB, are associated with reduced calcium absorption. We have studied the long-term impact of PPI upon calcium homeostasis among RYGB patients.

**Methods:**

In the Scandinavian Obesity Surgery Registry (SOReg), 550 primary RYGB patients, with eGFR > 60 mL/min/1.73 m^2^, had PTH and 25-OH D levels registered at 10 years. To avoid the impact of hypovitaminosis D, those with 25-OH D > 75 nmol/L were selected.

**Results:**

At 10 years, 10.3% of patients reported continuous PPI treatment, i.e., daily use during the last month. In an age adjusted logistic regression model, continuous PPI treatment was associated with a quadruple risk (OR: 4.65 [1.54–14.04]) of having a pathological PTH level (> 7 pmol/L).

**Conclusion:**

This unique study has shown a correlation between continuous PPI use and pathological PTH levels, thereby inferring that the medication may have detrimental effects upon calcium homeostasis among gastric bypass patients. The risk of having pathological PTH levels was more than tripled among those with PPI treatment, highlighting the importance of specialized follow-up while also suggesting that a limited duration of PPI treatment is preferable.

## Background

In parallel to the obesity epidemic, metabolic and bariatric surgery (MBS) has rocketed [1]. In a recent systematic review, MBS demonstrated a high remission rate of type 2 diabetes and a clinically significant decrease in other comorbidities. Furthermore, quality of life was improved [2]. Apart from massive weight loss, MBS is also a known cause of nutritional deficiencies. This creates a steady growth in the population in need of follow-up care.

The Roux-en-Y gastric bypass (RYGB) procedure, commonly used worldwide, entails altering the anatomy of the gastrointestinal tract, leading to a smaller gastric pouch and a bypass of the duodenum and proximal jejunum. Weight loss is achieved partially by rapidly reaching satiety and partially by hormonal mechanisms due to the altered intestinal passage of ingested nutrients. Simultaneously, the impaired absorption of vitamins is counteracted by regular peroral supplementation, with updated regimes for vitamin D, in both Sweden and internationally [3, 4]. Despite this, vitamin D deficiency and reduced calcium absorption are observed in patients after RYGB. Sequentially, vitamin D deficiency is a known cause of secondary hyperparathyroidism, which can be detrimental to bone health. Previous studies have shown an increase in PTH among RYGB patients [5]. In addition, a recent review reported an increased risk for fracture among bariatric patients [6].

Dyspepsia, often referred to as indigestion, is a common condition, in both patients with obesity and normal-weight individuals, with symptoms including heartburn and stomach pain [7]. One of the first lines of treatment includes a proton pump inhibitor (PPI), which reduces the amount of gastric acid produced in the stomach [8]. Several studies have established an association between PPI treatment and high PTH values among non-bariatric patients, although the underlying cause is still uncertain [9, 10]. Few studies to our knowledge have examined PTH levels among RYGB patients with regard to PPI treatment.

## Aim

This work aimed to study the impact that PPI medications have upon calcium homeostasis among RYGB patients long term.

## Materials and Method

Patient data was gathered from the Scandinavian Obesity Surgery Registry (SOReg), a national quality registry of MBS in Sweden since 2007. SOReg contains clinical and laboratory data at baseline and at 1-, 2-, 5-, and 10-year follow-up, all with high validity [11]. As of 2010 and 2012, respectively, data regarding vitamin supplementation, and PTH and 25-OH D levels could also be entered into the registry (not mandatory variables). We included patients who had undergone a primary RYGB, without reported revisions, with eGFR > 60 mL/min/1.73 m^2^, and had completed their 10-year postoperative follow-up. Patients without complete data on PTH and 25-OH D, those with 25-OH D < 75 nmol/L, or reported parathyroid surgery were excluded.

### Statistics

Data is presented as mean (± standard deviation). Means are compared using either the Student’s *t*-test for parametric data or Mann–Whitney *U* test for nonparametric data. Proportions are tested using Fischer’s exact test due to expected counts less than 5. A *p* < 0.05 was considered statistically significant. Regression models were calculated where PTH was defined as the dependent variable. Independent variables for all models were identified by correlation testing. A binomial logistic regression analysis was performed where the outcome was defined as normal PTH levels (PTH < 7.0 pmol/L) or pathological PTH levels (PTH $\ge$ 7 pmol/:). In this analysis, body mass index (BMI) and age were entered as continuous independent variables; however, 25-OH D was stratified as either below 100 nmol/L or 100 nmol/L and higher. The adjusted model was performed in a stepwise manner using independent variables that were significant from the unadjusted analyses. Statistical analysis was performed using the SPSS Statistics software (version 29.0); SPSS, Chicago, IL, USA).

## Results

Of the initial 1193 patients, 550 were excluded due to non-complete PTH and 25-OH D data, 467 due to 25-OH D < 75 nmol/L, and one because of parathyroid surgery, leaving a study population of 175 patients. Of those 162 who responded, 77.3% reported regular intake of dietary supplements containing additional vitamin D and calcium. At the 10-year postoperative follow-up, 10.3% of the included patients reported continuous PPI treatment, i.e., daily use during the last month. As presented in Table 1, significantly higher PTH levels were evident among those with ongoing PPI treatment, as were lower vitamin D levels.

**Table 1:** Comparison of patient demographics, PTH, and vitamin D levels in patients with and without daily use a mean of 10 years after RYGB

	PPI treatment (*n* = 18)	No PPI treatment (*n* = 157)	*p*-value
Men/women, %	16.7/83.3	19.8/80.2	1.00^A^
BMI at 10 years	32.3 ± 7.7	30.7 ± 5.6	0.385
Age	49.6 ± 12.8	53.4 ± 10.8	0.169
PTH	6.56 ± 2.55	5.15 ± 1.95	0.006
Vit D	84.2 ± 10.8	90.3 ± 15.2	0.049^B^

^A^Comparison via Fischer exact test

^B^Comparison via Mann–Whitney *U* test

**Fig. 1:**
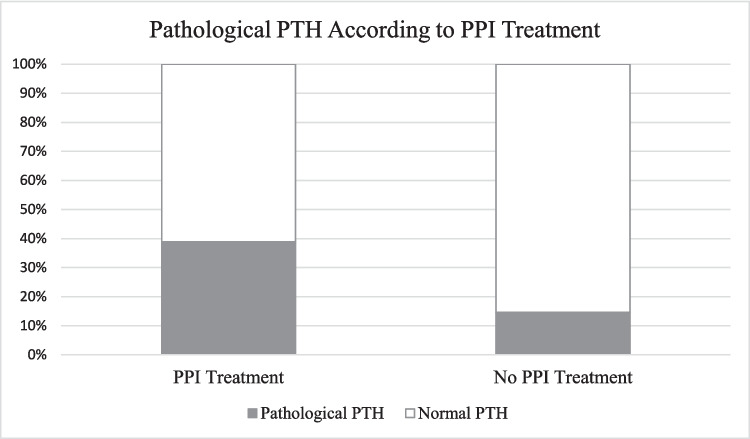
Proportion of patients with pathological PTH (PTH ≥ 7.0 pmol/L) depending upon PPI treatment

The proportion of patients with PTH levels at or above 7 pmol/L was significantly higher among patients with PPI treatment (*p* = 0.018) (Fig. 1).

Regular PPI treatment proved significant in both our simple and adjusted logistic regression model (OR 4.65 [1.54–14.04]) as did age (OR 1.05 [1.01–1.09]) (Table 2).

**Table 2:** Logistic regression model, pathological PTH as dependent variable

	Simple logistic regression				Adjusted logistic regression		
	Total (%)	Odds ratio	95% C.I	*p*-value	Odds ratio	95% C.I	*p*-value
PPI treatment							
No	157	Ref			Ref		
Yes	18	3.71	1.30–10.55	0.01	4.65	1.54–14.04	0.01
Age (years)	175	1.041	1.00–1.08	0.04	1.05	1.01–1.09	0.02
BMI (kg/m^2^)	175	1.073	1.01–1.14	0.03			
Sex							
Male	27	Ref					
Female	118	0.752	0.29–1.93	0.55			
Vit D (nmol/L)							
75–99	139	2.65	0.76–9.30	0.13			
100 and up	36	Ref					

## Discussion

Daily use of PPIs clearly has a measurable impact upon calcium homeostasis among RYGB patients, evident by the results of this study. Not only were the mean PTH values significantly higher but the odds of having pathological PTH levels were more than four times greater among those with PPI medications (Table 2), which is a clear sign of parathyroid activation.

Results from previous studies, including work done by our own group, have shown repeatedly that the RYGB procedure is associated with elevated PTH levels suggesting a negative impact upon calcium homeostasis [5, 12]. The fraction of calcium that is absorbed has been shown to decrease significantly after a RYGB procedure [13]. Consequentially, when adjusted for other known risk factors for high PTH levels (sex, age, BMI, and vitamin D levels), the procedure proves to be a significant factor in itself when predicting elevated PTH levels. Our previous work has also shown that with time since surgery, PTH levels continue to rise even when adjusted for age [12]. Conclusively, the procedure itself impedes calcium homeostasis which is mirrored by elevated PTH levels. This is presumably (at least in part) due to the excluded portion of the proximal intestine, which is a major site for vitamin D-dependent calcium absorption.

Studies have shown repeatedly that PPI therapy is associated with reduced calcium absorption [14–16]. Dating back to the 1950s, several reports of patients having undergone gastrectomies have shown that reduced gastric acid is linked to osteomalacia [17, 18]. Similarly, PPI treatment leads to a reduction in gastric acid, increased levels of gastrin, and hypochloridria, all of which are potential causes of elevated PTH levels [19]. A study conducted by Helgadottir showed increased gastrin levels among individuals taking PPI medications, regardless of type of PPI therapy, especially among women with increased BMI [20]. In line with the present study, Fitzpatrick and colleagues recently published a study reporting an OR of 1.6 for developing high PTH levels among non-bariatric patients medicating with PPIs [9]. The results from this study suggest an even greater risk among those having undergone RYGB as PPI treatment was associated with an OR of nearly five. A recent meta-analysis of non-operated individuals also reported a greater risk of fracture among those taking PPIs and concluded caution should be taken when prescribing to patients with other risk factors for osteoporosis [21]. Noteworthy, RYGB has repeatedly been identified as a major risk factor for osteoporosis [22]. Although RYGB results in a reduced rate of PPI treatment, it is still continuously used by roughly 10% of patients [23].

The altered anatomy following a RYGB leads to changes in the gastrointestinal environment. A previous study of the excluded stomach in RYGB patients, using a percutaneous gastrostomy, showed low acid output with simultaneous PPI treatment [24]. This implies reduced acidity along the proximal jejunum following the entero-entero anastomosis, which has been illustrated in other published research [3]. The reduced acidity likely alters the absorption of nutrients, including calcium, as observed in the aforementioned studies. This study underlines PPI treatment as an additional risk factor for pathological calcium homeostasis among RYGB patients.

The elevated PTH levels among RYGB patients are presumably caused by malabsorption and/or vitamin D deficiency, i.e., secondary hyperparathyroidism. It is however, beyond the scope of this study to specify due to insufficient data. Regardless, it is worth mentioning that secondary hyperparathyroidism is a silent state that can be detrimental to bone health when left untreated. Considering the impaired calcium homeostasis among RYGB patients, it is especially important to monitor for PTH pathology long term postoperatively and that this be taken into consideration when prescribing regular PPI treatment. Essentially, this stresses the need for long-term dedicated follow-up of these patients.

### Strengths and Limitations

The generalizability of the present results should be high as our patient cohort is very similar to the worldwide data presented in the last IFSO report (*n* = 480,970 procedures), e.g., proportion of female patients (80% vs. IFSO: 79 [range 60–86]%), median age at surgery (42 vs. 41 [31–45] years), and baseline BMI (42 vs 41 [36–45] kg/m^2^) [25].

Our study bases PPI treatment upon register data where patients responded whether they had regularly medicated for acid-related symptoms during the past month. Hence, patients with irregular PPI use are likely present in the “no treatment” group. In Sweden, like several other countries, PPI medications are available without a prescription. Thus, it is difficult to determine their use through patient charts and/or prescription registers, and patient reported use is likely a superior source of information. We have also not considered type of PPI nor dosage when drawing conclusions in this study. The fact that we could present significant results despite these drawbacks and the rather low number of patients implies a strong association between continuous PPI use and pathological PTH levels.

The lack of complete data regarding regular intake of dietary supplements is a limitation, as well as the lack of calcium levels halts the ability to decipher the type of pathological hyperparathyroidism observed in this population. However, removing those with low eGFR also excises the patients with elevated PTH levels due to kidney failure. In addition, the PTH levels in both groups, although at the higher end of the normal range, remained lower than levels typical for primary hyperparathyroidism.

## Conclusion

The results from this study has uniquely correlated continuous PPI use among RYGB patients as a risk factor for pathological PTH levels. This suggests that PPIs and the altered anatomy present a compounded threat to calcium homeostasis. Although the risk of having pathological PTH levels was more than tripled, patients with acid-related symptoms should not be denied PPIs, but the duration of treatment should be limited and specialized follow-up long term is key to reduce the risk of bone disease.

## Data Availability

No datasets were generated or analysed during the current study.
